# The Innovative Potential of Statins in Cancer: New Targets for New Therapies

**DOI:** 10.3389/fchem.2020.00516

**Published:** 2020-06-18

**Authors:** Elisabetta Di Bello, Clemens Zwergel, Antonello Mai, Sergio Valente

**Affiliations:** ^1^Department of Drug Chemistry and Technologies, Sapienza University of Rome, Rome, Italy; ^2^Department of Precision Medicine, Luigi Vanvitelli, University of Campania, Naples, Italy

**Keywords:** cancer, statins, drug combination, target therapy, mevalonate pathway, HMG-CoA reductase

## Abstract

Numerous and different types of cancers possess the dysregulation of the mevalonate pathway as a common feature. Statins, traditionally applied in cardiovascular diseases to reduce lipid levels, subsequently have been discovered to exhibit anti-cancer activities also. Indeed, statins influence proliferation, migration, and survival of cancer cells by regulating crucial signaling proteins, such as Rho, Ras, and Rac. Recently, several studies have demonstrated that simvastatin, fluvastatin, and lovastatin are implicated in different pathways that enhance the survival time of patients with cancer under treatment in combination with antineoplastic agents. In this minireview, we present an overview of the most important studies conducted regarding the use of statins in cancer therapy up to date.

## Introduction

To date, there are a lot of types of cancers described in the literature, with many associated treatments in constant evolution. In many forms of cancer, a dysregulation of the lipid metabolism (Fritz et al., 2013) and the mevalonate pathway (MVP) is observed (Freed-Pastor et al., 2012). Cholesterol is an indispensable component of cell membranes, and it is a precursor of bile acids, lipoproteins, and steroid hormones. Its biosynthesis is controlled by the MVP, which controls protein farnesylation and geranylation. These post-translational modifications are critical for the downstream signaling activity of Ras, Rho, or Rac proteins, that are part of the small GTPases superfamily (Takai et al., 2001), involved in tumorigenesis, progression (Buhaescu and Izzedine, 2007), proliferation, migration, and survival of tumor cells (Kidera et al., 2010). Like healthy cells, also cancer cells esterify fatty acids in phospholipids that are essential cell membrane components. These necessary lipids are obtained by the endogenous metabolites deriving from the MVP (Notarnicola et al., 2014). Inhibiting this vital process could be beneficial in cancer cells as they are usually rapidly proliferating without affecting too much healthy cells with a slower reproduction rate. Statins are able to decrease lipid levels in the plasma by inhibiting HMG-CoA reductase (HMGCR). Several studies have shown an intense correlation between the use of statins and cancer. First promising studies showed that statins are able to improve the outcome in cancer, i.e., prolonged the survival time (Gupta et al., 2019). In this minireview, we would like to illustrate new perspectives and innovative targets regarding the use of statins, such as simvastatin, fluvastatin, and lovastatin, in cancer alone or in combination with other drugs.

## Overview of Statins

**Figure 1:**
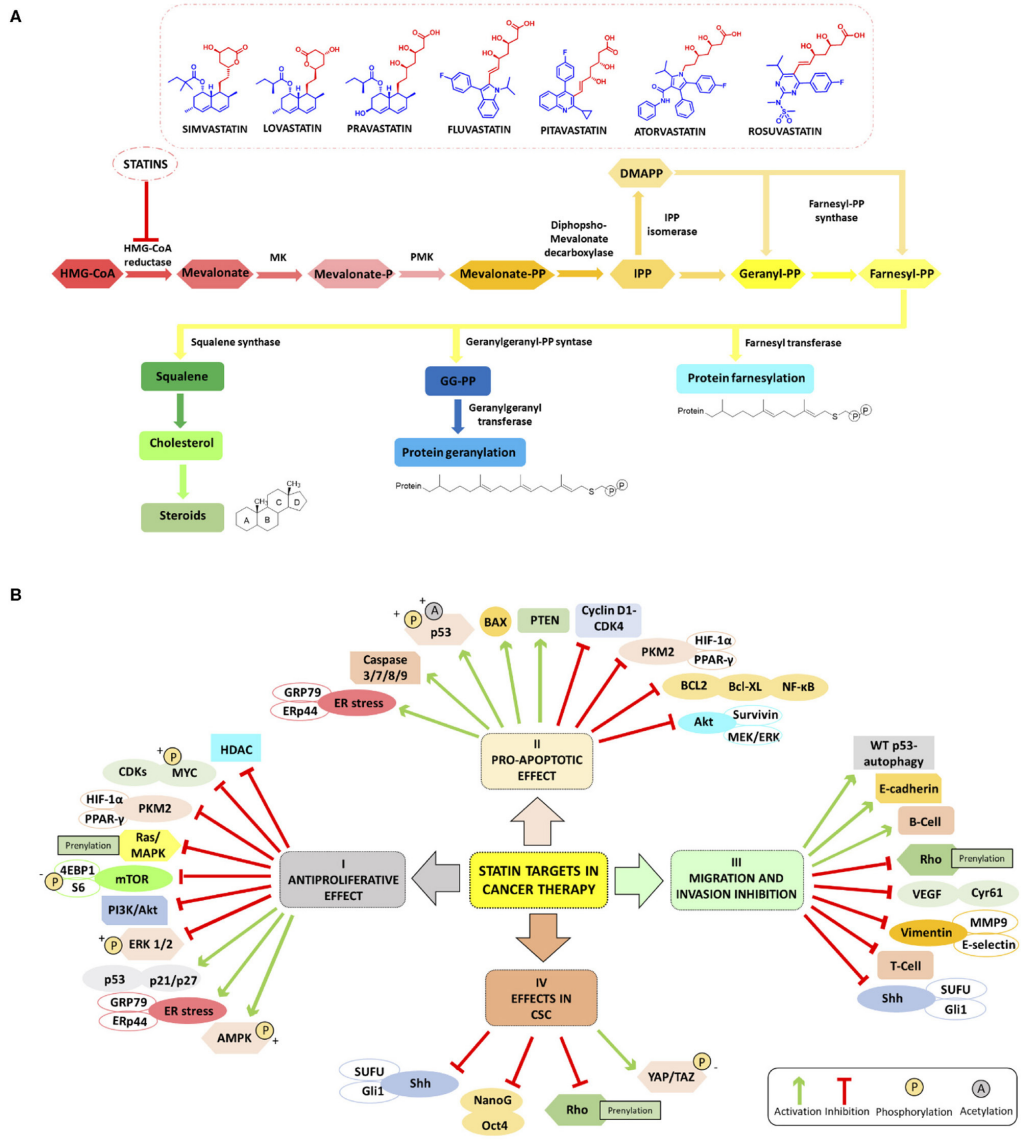
**(A)** Schematic summary of Mevalonate Pathway (MVP) and chemical structure of HMGCR inhibitors mainly studied in cancer (simvastatin, lovastatin, pravastatin, fluvastatin, pitavastatin, atorvastatin and rosuvastatin); the pharmacophore moiety in red and the ring system moiety in blue). **(B)** Overview of the main molecular targets of statins in cancer. I Antiproliferative effect: statins inhibit the proliferation of cancer cell increasing ER stress markers GRP78 and ERp44, phosphorylating S6 and 4EBP1 and inhibiting mTOR pathway (Okubo et al., 2020), and inhibiting HDACs (Lin et al., 2008; Chou et al., 2019). Statins involve LKB1-AMPK-p38MAPK-p53 survivin signaling cascade thanks to the phosphorylation and acetylation of p53 in cancer cells (Huang et al., 2020). II Pro-apoptotic effect: statins enhance apoptosis through inhibition of survivin protein expression (see above) (Huang et al., 2020), by inhibiting HIF-1α/PPAR-γ/PKM2-mediated glycolysis (Feng et al., 2020), induce caspase 3/7/8/9 protease, decrease the expression of cyclin D1 and CDK4 Bcl-XL (Okubo et al., 2020), and block the transcription factors NF-κB (Lee et al., 2014; Branvall et al., 2020). III Migration and invasion inhibition: statins induce T-cell suppression and B-cell survival (Branvall et al., 2020), and enhance the WT p53-dependent autophagy (Chou et al., 2019). IV Effects in CSC: statins can reduce the expression of Sonic Hedgehog (Shh) and consequently, the migration and invasion of cancer cells (Yin et al., 2018). Other cancerogenic pathways are reviewed in Iannelli et al. (2018).

The first statin, mevastatin, was identified and isolated by Endo (2004), being the first cholesterol-lowering drug. Today statins are classified into two groups: type-I derivatives are derived from fermentation products, such as mevastatin, lovastatin, pravastatin, and simvastatin. Type-II statins, including fluvastatin, atorvastatin, cerivastatin, pitavastatin, and rosuvastatin, are drugs of synthetic origin (Oryan et al., 2015). Statins are characterized by a characteristic pharmacophore group either being a lactone ring as a prodrug or a long chain carboxylic acid as an active form (red moiety in Figure 1A), responsible for the inhibiting activity, and a ring system moiety that is different for each type (blue moiety in Figure 1A) (Gazzerro et al., 2012). Lovastatin and simvastatin are lactones, thus closed ring prodrugs, which are transformed into their active open forms in the body; other statins are administered orally as active, opened rings, forming hydroxyl acids (Corsini et al., 1995) as their inhibitory effect on HMGCR is heavily depending on their solubility. However, one of the major limitations restricting the application of statins lies, for some of them, in the low bioavailability often below 50%. To improve bioavailability, nanocarriers loaded with statins, such as micelles, nanocrystals, and lipid-based nanoparticles (NPs), allowing a better drug uptake in the gastrointestinal tract, have been studied with promising results (Korani et al., 2019).

As already mentioned, statins are traditionally used for the reduction of lipid profiles in cardiovascular diseases (Goldstein and Brown, 2015). Mechanistically, statins inhibit HMGCR, and the depletion of intracellular cholesterol levels induces the expression of LDL receptors that decrease circulating LDL cholesterol in plasma, resulting in a decreased biosynthesis and increased cholesterol catabolism (Schonewille et al., 2016). For dyslipidemia, red yeast rice is used as a treatment option in traditional Chinese medicine as it contains a variable percentage of monacolin K which possesses actually the same chemical structure of lovastatin. However, red yeast rice, in lay literature often entitled as a superfood, should not be considered especially in cancer, as the monacolin K levels are not standardized. Besides the dosage problem, often carcinogens or other harmful by-products are found in this modified fermented rice, which make this “food” a risky choice (Dujovne, 2017). Statins have gained much attention for the prevention and treatment of cancer as they are capable to inhibit inflammation, angiogenesis, and proliferation and to induce apoptosis (Demierre et al., 2005). The antitumor effect of statins in monotherapy or in combination with anti-cancer agents is associated with cancer-related survival (Pisanti et al., 2014; Vallianou et al., 2014). Figure 1B summarizes the main molecular targets of statins in cancer. In the following paragraphs, we will give an overview of the main studies reported to date in the literature and the most innovative drug combinations that include both statins (simvastatin, lovastatin, pravastatin, fluvastatin, pitavastatin, atorvastatin, and rosuvastatin) and many types of anti-cancer agents endowed with antiproliferative, proapoptotic effects also able to impair migration and invasion processes in several cancers including cancer stem cells (CSCs).

## Simvastatin: the Most Investigated in Cancer Therapy

The rather lipophilic simvastatin has been shown in population-based cohort study to be more capable to reduct cancer-specific mortality than other hydrophilic statins. In a previous study, comparing atorvastatin, fluvastatin, lovastatin, pravastatin, rosuvastatin, and simvastatin, the latter one gave the best results regarding patients' survival (Cardwell et al., 2015). Recently, Chou et al. have revealed the role of statins in lung cancer. First, they retrospectively analyzed patients diagnosed with lung cancer between 1998 and 2011, and found a high percentage of patients with p53 mutations observing a reduced 5-years mortality under simvastatin treatment in this population-based study (Chou et al., 2019). The p53 gene is well-known tumor suppressor modulating numerous vital cellular functions, such as apoptosis, autophagy, senescence, often correlated with metabolic reprogramming (Bieging et al., 2014). In a second step, they wanted to verify the impact of p53 mutations upon treatment with simvastatin in lung adenocarcinoma cells (Chou et al., 2019).

In more detail, different types of cell lines (such as p53 wild type or p53 null) were used to study the cytotoxic effects of simvastatin in cell growth inhibition and decrease of lipid rafts in lung cancer cells with p53 mutations. Statins are likely to exhibit their antitumor effects (such as proliferation and migration impairment) by influencing inflammatory and oxidative stress-related tumorigenesis. Moreover, simvastatin inhibits tumor metastasis by WT p53-dependent autophagy. Statins have very likely tumor-suppressive effects in both normal and mutant p53 cells by regulating different signaling pathways (Chou et al., 2019).

Pancreatic Ductal Adenocarcinoma (PDA) is a disease with high therapy resistance, early metastasis, and poor prognosis (Siegel et al., 2014). An essential role for pancreatic CSCs is playing by Shh, which is a fundamental part of the Hedgehog signaling pathway, and whose expression is often highly upregulated in CSCs (Pak and Segal, 2016) affecting the survival of malignant cells. Moreover, Hedgehog signaling is depending on cholesterol and its levels (Porter et al., 1996). Yin et al. (2018) investigated if statins, especially simvastatin, could stop the progression of PDA. In their hands, simvastatin increased the efficacy of the chemotherapeutic agent gemcitabine in PDA cancer cells as well as in a chicken-egg xenograft model resulting in inhibited tumor growth, and reduced pancreatic CSC features acting on Shh signaling. Thus, this cancer-specific effect of simvastatin might be Hedgehog-dependent (Yin et al., 2018). Staying on this type of tumor, McGregor and colleagues demonstrated *in vitro* and *in vivo* that PDA depends on MVP for *de novo* synthesizing Coenzyme Q (CoQ) (McGregor et al., 2020). They showed *in vitro* a synergistic interaction between simvastatin and the mitogen-activated protein kinase (MEK) inhibitor AZD6244. However, CoQ is available from the diet and might not be limited in the context of *in vivo* physiology. The authors further verified their results in a KPC PDA mouse model, and they observed that statin treatment induced elevated ROS production, and this increase was mitigated by cancer cells through upregulation of the antioxidant metabolic pathways. When the mice were treated with both statin and the MEK inhibitor AZD6244, there was a significant increase in apoptosis and cell death through elevated ROS production.

Hepatocellular carcinoma (HCC), a frequently occurring primary malignant tumors worldwide (Bray et al., 2018), is associated with delays in diagnosis and a high tendency to metastasis development (Niu et al., 2017). Sorafenib is used in cancer therapy since it possesses antiproliferative effects via the inhibition of kinases, such as CRAF, BRAF, and c-KIT and moreover is able to reduce angiogenesis through the inhibition of vascular endothelial growth factor 2/3 (VEGFR-2/3). Feng et al. (2020) have studied the problem of sorafenib resistance in HCC, demonstrating that simvastatin is able to counteract this resistance when administered in combination with sorafenib. They have used LM3, and sorafenib-resistant LM3 (LM3-SR) cell lines to study cell proliferation, apoptosis and glycolysis levels, and afterwards, they translated both cell lines into xenograft mouse models using oral formulations containing sorafenib and simvastatin alone or in combination. Their results have shown that the suppression of HIF-1α/PPAR-γ/PKM2 axis by simvastatin augments the sensitivity of LM3-SR cells to sorafenib when combined with simvastatin. Furthermore, this treatment decreased proliferation and increased apoptosis in HCC cells.

Moreover, about HCC, the cyclooxygenase-2 (COX-2) has been shown to be overexpressed in this type of cancer (Shiota et al., 1999). The primary metabolite of COX-2 is prostaglandin E2 and is associated with cell proliferation and resistance to apoptosis, thus the use of celecoxib, a COX-2 specific inhibitor, might result in the inhibition of HCC cell growth (Gao et al., 2010). Lee et al. supposed that the combination between simvastatin and NS398, a COX-2 inhibitor, could maximize pharmacological efficacy and may minimize adverse effects in HCC exhibiting a synergistic effect in Hep3B and Huh-7 cell lines. The aforementioned drug combination was shown to suppress the nuclear localization of nuclear factor-kappa-B (NF-κB), to inhibit the Akt pathway, to decrease procaspase 3 and Bcl-2 levels, and to augment caspase 9 levels, being more effective than the single treatments (Lee et al., 2014).

The NF-κB pathway is also upregulated in multiple myeloma (MM) cells, and it may be targeted in novel, innovative MM treatments (Annunziata et al., 2007). As we have seen in HCC, statins might lead to a downregulation of the NF-κB pathway, thus underpinning the probable positive outcomes in MM patients under statin treatment (Martin-Ventura et al., 2005). Brånvall et al. used a Swedish population-based national health register and performed a follow-up study on 4,315 MM cases. Various statins were followed up, but simvastatin was the most widely used. Some patients received no statins while others received them during the 6-months period preceding the MM diagnosis, and the last group during 6 months after diagnosis. The analysis revealed that the statin use before and after the diagnosis was linked to a reduced risk of mortality in both sexes (Branvall et al., 2020). The authors suppose in their discussion that the observed positive effects of statins are connected with a promotion of the host immune response by regulating mesenchymal stromal cell-induced T-cell suppression and B-lymphocyte survival as earlier described *in vitro* (Musso et al., 2011). The study mentioned above represents an interesting shred of evidence regarding the use of statins in MM, warranting further *in vitro* and *in vivo* experiments.

Simvastatin has also been tested to check its putative influence in Epithelial-Mesenchymal Transition (EMT). The transforming growth factor-β1 (TGF-β1) is a key inducer and driver of the EMT process. Yang et al. (2013) observed that simvastatin could attenuate TGF-β1-induced EMT in human alveolar A549 cells but failed to reverse the cell morphological changes. Despite the somewhat discouraging results, further research efforts are indispensable to dissect better the role of statins in lung cancer.

## Statins and Epigenetics

Epigenetics mechanisms, being up today strongly validated in cancer pathogenesis (see for details the following reviews) (Jones et al., 2016; Flavahan et al., 2017; Mai, 2020), have been recently studied even among the different biological effects of statins, and the role of the latter in epigenetics is more and more evolving.

Human tumors are frequently characterized by dysregulation between the activities of histone acetyltransferases (HATs) and histone deacetylases (HDACs) (Fraga et al., 2005). For instance, the acetylation of lysine residues on histone H3 and H4 increases the expression of p21, thus activating the tumor suppressor p53. Many pieces of evidence are showing that non-lipid effects by statins play a pivotal role in their anti-cancer activity (Wong et al., 2002). Lin et al. have compared the molecular structures of HDAC inhibitors (like vorinostat or TSA) and statins: both of them contain acidic moieties, such as hydroxamic and carboxylic acid functions, respectively, and they have discovered that statins probably induce hyperacetylation of H3 thanks to the inhibition of HDAC1/2. They also have performed molecular docking studies assessing that the carboxylic acid moiety of statins is able to enter the catalytic site of the human HDAC2. Next, the inhibition of HDAC by lovastatin, atorvastatin, pravastatin, and simvastatin was determined by using the Fluor-de-Lys HDAC activity assay kit. They have shown the dissociation of HDAC1/2 and the association of CREB binding protein (CBP) at the p21 promoter eliciting p21 expression and increased histone-H3 acetylation levels. The statins above mentioned were able to decrease the cell proliferation and tumor growth in several cellular models, such as lung cancer (A549, NCI-H292), colorectal cancer (HCT-116, HCT-116 p53), and AGS human gastric carcinoma cells, and in a xenograft female BALB/c nude mouse model of A549 lung cancer (Lin et al., 2008).

Recently, it has been shown by Okubo et al. (2020) that combinations of fluvastatin and vorinostat act cooperatively against renal cancer cells. In more detail, the anti-cancer activity of the HDAC inhibitor vorinostat was less evident because of the intrinsic activation of the mTOR pathway in a renal cancer cell model. The authors wanted to overcome this undesired activation through a co-treatment with fluvastatin, which is known to activate the AMP-activated protein kinase (AMPK) as a mTOR inhibitor. This is of particular interest because AMPK controls cellular metabolism pathways essential for cancer progression, thus being a vital regulator of cancer cell growth and proliferation (Faubert et al., 2015). The authors were able to demonstrate that the combination of vorinostat and fluvastatin enhanced the vorinostat-induced histone acetylation, suppressed the vorinostat-activated mTOR pathway via pivotal fluvastatin-related AMPK activation, thus resulting in renal anti-cancer effect *in vitro* (human renal cancer cells ACHN, A498, murine renal cancer Renca) and *in vivo* (Renca mouse model) via apoptosis induction. Noteworthy, the phosphorylation of downstream protein of the mTOR pathway 4EBP1 is decreased by vorinostat and fluvastatin co-treatment, and it reduced renal cancer viability. The results they have achieved indicate that the vorinostat-fluvastatin combination would be a safe and effective combination therapy warranting further examination.

These promising results might pave the way toward innovative cancer therapies and/or chemoprevention strategies. HDAC inhibitors and statins used alone do not give excellent results, and they are characterized by side effects or lower bioavailability. In 2013, Chen and colleagues designed some innovative dual-action inhibitors to target HDAC as well as HMGCR. Interestingly, the compounds showed potent inhibitory activities with IC_50_ values in the nanomolar range, via the presence of the two essential pharmacophoric elements: hydroxamic acid moiety responsible for the HDAC inhibition and dihydroxy-hydroxamate moiety responsible for the HMGCR inhibition. They used molecular docking to verify the affinity of their hybrid molecules to HDAC and HMGCR; then they performed *in vitro* studies to examine enzyme inhibition activity as well as cell growth inhibition in A549 human lung cancer cells, MEF normal mouse and HS68 human fibroblasts. Some of their dual-inhibitors possess a good selectivity for cancer cells when compared with healthy ones (Chen et al., 2013). The compounds mentioned above were studied further by the same research group in more detail. Wei et al. (2016a) tested the molecules in C57BL/6, BALB/c, NOD/SCID mice, and Apc(Min/+) colorectal cancer (CRC) mouse models obtaining inhibition of metastasis, angiogenesis, and cancer stemness; furthermore, such compounds boosted the effect of oxaliplatin in the aforementioned CRC mouse models. The potential benefits of the dual targeting HDAC/HMGCR inhibitor has been confirmed in a subsequent study of the same research team underlining the potential benefits of this promising lead compound able to prevent colitis-associated colorectal cancer (Wei et al., 2016b). Next, Weng et al. (2019) were able to demonstrate in a non-small lung cancer cell (NSCLC) model, the most common of lung cancers, that the hybrids are able to inhibit the EMT. This pivotal study underlines the feasibility of dual-targeting anti-cancer agents based on HDAC and statin moieties.

## Non-epigenetic Pathways Modulated by Statins

Otahal et al. demonstrated *in vitro* the effectiveness of the combination of fluvastatin or pitavastatin with erlotinib, an approved EGFR tyrosine kinase inhibitor (EGFR-TKI), in EGFR-TKI resistant human lung adenocarcinoma cell lines with mutated or overexpressed EGFR, such as A549, Calu6, and H1993, exhibiting promising cytotoxic effects. They proved that both statins combined with erlotinib led to apoptosis in NSCLC cell lines *in vitro*, particularly in K-Ras mutated cell lines (Otahal et al., 2020).

Regarding cancer-related deaths in women, breast cancer is one of the leading causes (Siegel et al., 2017). This cancer needs new strategies and/or new treatments due to its high mortality and aggressiveness. Survivin, the smallest member of the inhibitors of apoptosis protein (IAP) family, modulates numerous cellular processes for example mitosis, migration, angiogenesis or chemo-resistance. Recent studies demonstrated that the use of statins reduces breast cancer-specific mortality in patients (Cardwell et al., 2015; Liu et al., 2017). Huang et al. have highlighted that lovastatin could have a beneficial effect in various cell models, such as MCF-7, MDA-MB-231, and T47D. They showed that lovastatin inhibited cell proliferation and induced apoptosis in MCF-7 cells by causing p21 expression, thus leading to p53 level increase and survivin downregulation. The mechanism is not yet fully elucidated, but lovastatin exhibits its antitumor activities via the LKB1-AMPK-p38MAPK-p53 survivin cascade resulting in survivin reduction and ultimately cell death, thus suggesting an important role of this statin for breast cancer treatment (Huang et al., 2020). However, the precise mechanism by which lovastatin causes cell death needs to be studied more in-depth and probably depends on tumor subtypes and/or the patient's genetic background.

C-Myc, an important transcription factor regulating crucial biological cell functions, including cell cycle, differentiation and proliferation, is often deregulated in cancer through somatic mutation, chromosomal translocation, genomic amplification of defects in upstream regulators (Albihn et al., 2010). C-Myc is overexpressed in 31–64% of medulloblastoma types (Eberhart et al., 2004). MiR-33b is a specific inhibitor of c-Myc, which is frequently lost in medulloblastoma. Its overexpression leads to the down-regulation of c-Myc and its transactivation targets, such as cyclin E or ornithine decarboxylase (ODC). Takwi et al. (2012) have proven that lovastatin upregulated the expression of mi-R-33b, leading to reduced cell proliferation. Lovastatin treatment also impairs orthotopically xenografted cells tumor growth. This study could be important to purpose statins as a pharmacological modulator of c-Myc via miRNA-based therapeutics.

Jiao et al. (2020) discovered that the treatment with pitavastatin of human MCF10A exhibiting oncogenic defects, such as cells lacking PTEN or expressing K-Ras^G12V^, led to geranylgeranyl diphosphate (GGPP) depletion of the MVP, to amino acid starvation and ultimately to cell death.

## Concluding Remarks

To sum up, statins have been used as lipid-lowering drugs to treat hyperlipidemia and to decrease cholesterol levels in the blood. It is now well-established that the antitumor effects of statins can be associated with MVP-mediated and non-MVP-mediated mechanisms (Figure 1). The MVP-pathway is interrupted at the first step through statin treatment that inhibits HMGCR halting the isoprenoid synthesis, such as GGPP and farnesyl diphosphate (FPP) for GTPase-proteins vital for cancer cells. Statins are regulators of the proliferation, migration, and survival of tumor cells by regulating Rho, Ras, and Rac proteins. Statins can also inhibit cancer cell growth by modulating specific other pathways. For example, very recently lovastatin was demonstrated to activate the LKB1-AMPK-p38MAPK-p53-survivin cascade causing MCF-7 breast cancer cell death (Huang et al., 2020). The simultaneous administration of fluvastatin and vorinostat effectively led to apoptosis and reduced renal cancer growth *in vitro* and *in vivo* through AMPK activation, histone acetylation, and ER stress induction (Okubo et al., 2020). Simvastatin is capable to inhibit the HIF-1α/PPAR-γ/PKM2 axis via the suppression of PKM2-mediated glycolysis, leading to a decreased proliferation and an increased apoptosis rate in HCC cells. Furthermore, HCC cell are resensitized to sorafenib treatment (Feng et al., 2020). Simvastatin treatment reduced CoQ synthesis and promoted oxidative stress and apoptosis in tumors when administered in combination with the MEK inhibitor AZD6244, highlighting a new mechanism through which statin treatment may impact PDA cancer growth (McGregor et al., 2020). Simvastatin led to mutant p53 protein degradation by activating a caspase-dependent apoptotic pathway and decreased motility in lung cancer cells possessing p53 missense mutations (Chou et al., 2019). The same drug hampered viability, stemness, tumor growth, and metastasis in pancreatic cells, via the inhibition of the Shh signaling leading to enhanced efficacy of gemcitabine treatment (Yin et al., 2018).

In this minireview, we have seen that statins are low cost drugs with few side effects that can be applied in numerous types of cancer. At present, in numerous clinical trials statins are investigated either alone or in combination with other anti-cancer agents, and an overview of the main ones is described in Table 1.

**Table 1:** Summary of the main clinical trials of statins investigated in cancer therapy.

**Study**	**Phase, trial number, start date**	**Disease(s)**	**Drug(s)**	**Sponsor**	**Status**
Simvastatin plus dual AntiHER2 therapy for metastatic breast cancer	II NCT03324425, 06/2019	Breast Cancer Stage IV	Simvastatin	Baylor Breast Care Center	Recruiting
Atorvastatin in treating patients with stage IIb–III triple negative breast cancer who did not achieve a pathologic complete response after receiving neoadjuvant chemotherapy	II NCT03872388, 01/2019	Triple Negative Breast Cancer	- Atorvastatin - Capecitabine	- M.D. Anderson Cancer Center - National Cancer Institute (NCI)	Recruiting
Survival benefits of statins in breast cancer patients	III NCT03971019, 03/2018	Breast Cancer	Statins	Peking Union Medical College Hospital	Recruiting
The effect of simvastatin on breast cancer cell growth in women with stage I–II breast cancer	II NCT03454529, 03/2018	Breast Cancer	Simvastatin	- National Cancer Institute (NCI) - Barbara Ann Karmanos Cancer Institute	Recruiting
Neoadjuvant zoledronate and atorvastatin in triple negative breast cancer	II NCT03358017, 03/2018	Triple Negative Breast Cancer	- Zoledronate - Atorvastatin	- Mario Negri Institute for Pharmacological Research - Associazione Italiana per la Ricerca sul Cancro	Recruiting
Effect of GDC-0810 on the pharmacokinetics of pravastatin in healthy female subjects of non-childbearing potential	I NCT02621957, 12/2015	Breast Cancer	- GDC-0810 - Pravastatin	Genentech, Inc.	Completed
Lipitor and biGuanide to androgen delay trial	II NCT02497638, 06/2020	Prostate Cancer	- Metformin - Atorvastatin - Placebo	University Health Network, Toronto	Not yet recruiting
Impact of atorvastatin on prostate cancer progression during ADT	III NCT04026230, 08/2019	Prostate Cancer	- Atorvastatin - Placebo	- Tampere University Hospital - Turku University Hospital - Central Finland Hospital District - Tartu University Hospital - University of Aarhus - Fimlab laboratories - Helsinki University Central Hospital - Kuopio University Hospital	Recruiting
Trial of acetylsalicylic acid and atorvastatin in patients with castrate-resistant prostate cancer	III NCT03819101, 03/2019	Prostate Cancer	- Acetylsalicylic acid - Atorvastatin	- Gustave Roussy, Cancer Campus, Grand Paris - National Cancer Institute, France	Not yet recruiting
Cellular effect of cholesterol lowering prior to prostate removal	I NCT02534376, 09/2015	Prostate Cancer	- Ezetimibe - Simvastatin	- Cedars-Sinai Medical Center - Roswell Park Cancer Institute	Completed
Atorvastatin before prostatectomy and prostate cancer	II NCT01821404, 08/2012	Prostate Cancer	Atorvastatin - Placebo	- Tampere University Hospital - Tampere University - Fimlab laboratories - University of Eastern Finland - Finnish Cultural Foundation	Completed
Statin combination therapy in patients receiving sorafenib for advanced hepatocellular carcinoma	II NCT03275376, 12/2017	Hepatocellular Carcinoma	- Atorvastatin - Placebo	Taichung Veterans General Hospital	Recruiting
Statin for preventing hepatocellular carcinoma recurrence after curative treatment	IV NCT03024684, 01/2017	Hepatocellular Carcinoma	- Atorvastatin - Placebo	- Chiayi Christian Hospital - E-DA Hospital - National Taiwan University Hospital - Taichung Veterans General Hospital - Mackay Memorial Hospital - Tainan Municipal Hospital - National ChengKung University Hospital - Chi Mei Medical Hospita	Recruiting
A single arm, phase II study of simvastatin plus XELOX and bevacizumab as first-line chemotherapy in metastatic colorectal cancer patients	II NCT02026583, 12/2013	Colorectal Cancer	Simvastatin	Samsung Medical Center	Completed
Rosuvastatin in treating patients with stage I or stage II colon cancer that was removed by surgery	III NCT01011478, 03/2010	Colorectal Cancer	- Rosuvastatin - Placebo	- NSABP Foundation Inc - National Cancer Institute (NCI)	Terminated
A phase I study of high dose simvastatin in patients with gastrointestinal tract cancer who failed to standard chemotherapy	I NCT03086291, 01/2018	Stomach Cancer	Simvastatin	Samsung Medical Center	Recruiting
Monitoring and treatment of relapsed leukemia following allogeneic hematopoietic stem cell transplantation in children	I NCT02484261, 05/2015	Leukemia	- Bortezomib - Pravastatin	- Reuven Schore - Millennium Pharmaceuticals, Inc. - Hyundai Hope On Wheels - Children's National Research Institute	Active, not recruiting
Efficacy and safety of atorvastatin in combination with radiotherapy and temozolomide in glioblastoma	II NCT02029573, 01/2014	Glioblastoma Multiforme	- Atorvastatin - Temozolomide - Radiotherapy	King Fahad Medical City	Completed
A pre-op window study evaluating anti-proliferative effects of atorvastatin on the endometrium	I NCT02767362, 11/2015	Endometrial Cancer	Atorvastatin	- UNC Lineberger Comprehensive Cancer Center - Wilma Williams Education and Clinical Research for Endometrial Cancer Award	Active, not recruiting
Simvastatin effect on radiation therapy of brain metastases	II NCT02104193, 04/2014	Brain Metastases	Simvastatin + Radiation therapy	Ain Shams University	Completed
Sorafenib tosylate with or without pravastatin in treating patients with liver cancer and cirrhosis	III NCT01075555, 02/2010	Liver Cancer	- Pravastatin Sodium - Sorafenib Tosylate	- Federation Francophone de Cancerologie Digestive - Center Hospitalier Universitaire Dijon	Completed

These studies are in constant evolution, and we can expect new combination treatments to come in the near future. Scientists could probably step up and develop hybrid inhibitors, confirmed to be a feasible approach as in the pivotal study of Chen et al. (2013), to further enhance the anti-cancer activity and decrease the side effects. However, despite the promising results achieved by the treatment with statins alone or in combination with other anti-cancer agents, the precise mechanisms how they exert their antitumor effects often remain unclear. In the years to come, we can expect novel and promising approaches addressing the statins to fight cancer, probably also at clinical levels.

## Author Contributions

ED and CZ performed the literature research and drafted a first version of the manuscript. AM and SV supervised and coordinated the work as well as corrected the manuscript.

## Conflict of Interest

The authors declare that the research was conducted in the absence of any commercial or financial relationships that could be construed as a potential conflict of interest.

## References

[B1] Albihn A. Johnsen J. I. Henriksson M. A. (2010). MYC in oncogenesis and as a target for cancer therapies. Adv. Cancer Res. 107, 163–224. 10.1016/S0065-230X(10)07006-5 20399964

[B2] Annunziata C. M. Davis R. E. Demchenko Y. Bellamy W. Gabrea A. Zhan F.. (2007). Frequent engagement of the classical and alternative NF-kappaB pathways by diverse genetic abnormalities in multiple myeloma. Cancer Cell 12, 115–130. 10.1016/j.ccr.2007.07.004 17692804 PMC2730509

[B3] Bieging K. T. Mello S. S. Attardi L. D. (2014). Unravelling mechanisms of p53-mediated tumour suppression. Nat. Rev. Cancer 14, 359–370. 10.1038/nrc3711 24739573 PMC4049238

[B4] Branvall E. Ekberg S. Eloranta S. Wasterlid T. Birmann B. M. Smedby K. E. (2020). Statin use is associated with improved survival in multiple myeloma: a Swedish population-based study of 4315 patients. Am. J. Hematol. 95, 652–661. 10.1002/ajh.25778 32141627

[B5] Bray F. Ferlay J. Soerjomataram I. Siegel R. L. Torre L. A. Jemal A. (2018). Global cancer statistics 2018: GLOBOCAN estimates of incidence and mortality worldwide for 36 cancers in 185 countries. CA Cancer J. Clin. 68, 394–424. 10.3322/caac.21492 30207593

[B6] Buhaescu I. Izzedine H. (2007). Mevalonate pathway: a review of clinical and therapeutical implications. Clin. Biochem. 40, 575–584. 10.1016/j.clinbiochem.2007.03.016 17467679

[B7] Cardwell C. R. Mc Menamin U. Hughes C. M. Murray L. J. (2015). Statin use and survival from lung cancer: a population-based cohort study. Cancer Epidemiol. Biomarkers Prev. 24, 833–841. 10.1158/1055-9965.EPI-15-0052 25934831

[B8] Chen J. B. Chern T. R. Wei T. T. Chen C. C. Lin J. H. Fang J. M. (2013). Design and synthesis of dual-action inhibitors targeting histone deacetylases and 3-hydroxy-3-methylglutaryl coenzyme a reductase for cancer treatment. J. Med. Chem. 56, 3645–3655. 10.1021/jm400179b 23570542

[B9] Chou C. W. Lin C. H. Hsiao T. H. Lo C. C. Hsieh C. Y. Huang C. C.. (2019). Therapeutic effects of statins against lung adenocarcinoma via p53 mutant-mediated apoptosis. Sci. Rep. 9:20403. 10.1038/s41598-019-56532-6 31892709 PMC6938497

[B10] Corsini A. Maggi F. M. Catapano A. L. (1995). Pharmacology of competitive inhibitors of HMG-CoA reductase. Pharmacol. Res. 31, 9–27. 10.1016/1043-6618(95)80042-5 7784310

[B11] Demierre M. F. Higgins P. D. Gruber S. B. Hawk E. Lippman S. M. (2005). Statins and cancer prevention. Nat. Rev. Cancer 5, 930–942. 10.1038/nrc1751 16341084

[B12] Dujovne C. A. (2017). Red yeast rice preparations: are they suitable substitutions for statins? Am. J. Med. 130, 1148–1150. 10.1016/j.amjmed.2017.05.013 28601545

[B13] Eberhart C. G. Kratz J. Wang Y. Summers K. Stearns D. Cohen K.. (2004). Histopathological and molecular prognostic markers in medulloblastoma: c-myc, N-myc, TrkC, and anaplasia. J. Neuropathol. Exp. Neurol. 63, 441–449. 10.1093/jnen/63.5.441 15198123

[B14] Endo A. (2004). The origin of the statins. Atheroscler. Suppl. 5, 125–130. 10.1016/j.atherosclerosissup.2004.08.033 15531285

[B15] Faubert B. Vincent E. E. Poffenberger M. C. Jones R. G. (2015). The AMP-activated protein kinase (AMPK) and cancer: many faces of a metabolic regulator. Cancer Lett. 356, 165–170. 10.1016/j.canlet.2014.01.018 24486219

[B16] Feng J. Dai W. Mao Y. Wu L. Li J. Chen K.. (2020). Simvastatin re-sensitizes hepatocellular carcinoma cells to sorafenib by inhibiting HIF-1alpha/PPAR-gamma/PKM2-mediated glycolysis. J. Exp. Clin. Cancer Res. 39:24. 10.1186/s13046-020-1528-x 32000827 PMC6993409

[B17] Flavahan W. A. Gaskell E. Bernstein B. E. (2017). Epigenetic plasticity and the hallmarks of cancer. Science 357:eaal2380. 10.1126/science.aal2380 28729483 PMC5940341

[B18] Fraga M. F. Ballestar E. Villar-Garea A. Boix-Chornet M. Espada J. Schotta G.. (2005). Loss of acetylation at Lys16 and trimethylation at Lys20 of histone H4 is a common hallmark of human cancer. Nat. Genet. 37, 391–400. 10.1038/ng1531 15765097

[B19] Freed-Pastor W. A. Mizuno H. Zhao X. Langerod A. Moon S. H. Rodriguez-Barrueco R.. (2012). Mutant p53 disrupts mammary tissue architecture via the mevalonate pathway. Cell 148, 244–258. 10.1016/j.cell.2011.12.017 22265415 PMC3511889

[B20] Fritz V. Benfodda Z. Henriquet C. Hure S. Cristol J. P. Michel F.. (2013). Metabolic intervention on lipid synthesis converging pathways abrogates prostate cancer growth. Oncogene 32, 5101–5110. 10.1038/onc.2012.523 23208508 PMC3806338

[B21] Gao J. Jia W. D. Li J. S. Wang W. Xu G. L. Ma J. L.. (2010). Combined inhibitory effects of celecoxib and fluvastatin on the growth of human hepatocellular carcinoma xenografts in nude mice. J. Int. Med. Res. 38, 1413–1427. 10.1177/147323001003800423 20926014

[B22] Gazzerro P. Proto M. C. Gangemi G. Malfitano A. M. Ciaglia E. Pisanti S.. (2012). Pharmacological actions of statins: a critical appraisal in the management of cancer. Pharmacol. Rev. 64, 102–146. 10.1124/pr.111.004994 22106090

[B23] Goldstein J. L. Brown M. S. (2015). A century of cholesterol and coronaries: from plaques to genes to statins. Cell 161, 161–172. 10.1016/j.cell.2015.01.036 25815993 PMC4525717

[B24] Gupta A. Stokes W. Eguchi M. Hararah M. Amini A. Mueller A.. (2019). Statin use associated with improved overall and cancer specific survival in patients with head and neck cancer. Oral Oncol. 90, 54–66. 10.1016/j.oraloncology.2019.01.019 30846177 PMC6659746

[B25] Huang S. W. Chyuan I. T. Shiue C. Yu M. C. Hsu Y. F. Hsu M. J. (2020). Lovastatin-mediated MCF-7 cancer cell death involves LKB1-AMPK-p38MAPK-p53-survivin signalling cascade. J. Cell. Mol. Med. 24, 1822–1836. 10.1111/jcmm.14879 31821701 PMC6991643

[B26] Iannelli F. Lombardi R. Milone M. R. Pucci B. De Rienzo S. Budillon A. (2018). Targeting mevalonate pathway in cancer treatment: repurposing of statins. Recent. Pat. Anticancer. Drug. Discov. 13, 184–200. 10.2174/1574892812666171129141211. 29189178

[B27] Jiao Z. Cai H. Long Y. Sirka O. K. Padmanaban V. Ewald A. J.. (2020). Statin-induced GGPP depletion blocks macropinocytosis and starves cells with oncogenic defects. Proc. Natl. Acad. Sci. U.S.A. 117, 4158–4168. 10.1073/pnas.1917938117 32051246 PMC7049144

[B28] Jones P. A. Issa J. P. Baylin S. (2016). Targeting the cancer epigenome for therapy. Nat. Rev. Genet. 17, 630–641. 10.1038/nrg.2016.93 27629931

[B29] Kidera Y. Tsubaki M. Yamazoe Y. Shoji K. Nakamura H. Ogaki M.. (2010). Reduction of lung metastasis, cell invasion, and adhesion in mouse melanoma by statin-induced blockade of the Rho/Rho-associated coiled-coil-containing protein kinase pathway. J. Exp. Clin. Cancer Res. 29:127. 10.1186/1756-9966-29-127 20843370 PMC2949822

[B30] Korani S. Korani M. Bahrami S. Johnston T. P. Butler A. E. Banach M.. (2019). Application of nanotechnology to improve the therapeutic benefits of statins. Drug Discov. Today 24, 567–574. 10.1016/j.drudis.2018.09.023 30292917

[B31] Lee S. J. Hwang J. W. Yim H. Yim H. J. Woo S. U. Suh S. J.. (2014). Synergistic effect of simvastatin plus NS398 on inhibition of proliferation and survival in hepatocellular carcinoma cell line. J. Gastroenterol. Hepatol. 29, 1299–1307. 10.1111/jgh.12503 24372723

[B32] Lin Y. C. Lin J. H. Chou C. W. Chang Y. F. Yeh S. H. Chen C. C. (2008). Statins increase p21 through inhibition of histone deacetylase activity and release of promoter-associated HDAC1/2. Cancer Res. 68, 2375–2383. 10.1158/0008-5472.CAN-07-5807 18381445

[B33] Liu B. Yi Z. Guan X. Zeng Y. X. Ma F. (2017). The relationship between statins and breast cancer prognosis varies by statin type and exposure time: a meta-analysis. Breast Cancer Res. Treat 164, 1–11. 10.1007/s10549-017-4246-0 28432513

[B34] Mai A. (2020). Chemical epigenetics. Topics Med. Chem. 33:567 10.1007/978-3-030-42982-9

[B35] Martin-Ventura J. L. Blanco-Colio L. M. Gomez-Hernandez A. Munoz-Garcia B. Vega M. Serrano J.. (2005). Intensive treatment with atorvastatin reduces inflammation in mononuclear cells and human atherosclerotic lesions in one month. Stroke 36, 1796–1800. 10.1161/01.STR.0000174289.34110.b0 16020773

[B36] McGregor G. H. Campbell A. D. Fey S. K. Tumanov S. Sumpton D. Blanco G. R.. (2020). Targeting the metabolic response to statin-mediated oxidative stress produces a synergistic antitumor response. Cancer Res. 80, 175–188. 10.1158/0008-5472.CAN-19-0644 31562248

[B37] Musso A. Zocchi M. R. Poggi A. (2011). Relevance of the mevalonate biosynthetic pathway in the regulation of bone marrow mesenchymal stromal cell-mediated effects on T-cell proliferation and B-cell survival. Haematologica 96, 16–23. 10.3324/haematol.2010.031633 20884711 PMC3012760

[B38] Niu L. Liu L. Yang S. Ren J. Lai P. B. S. Chen G. G. (2017). New insights into sorafenib resistance in hepatocellular carcinoma: responsible mechanisms and promising strategies. Biochim. Biophys. Acta Rev. Cancer 1868, 564–570. 10.1016/j.bbcan.2017.10.002 29054475

[B39] Notarnicola M. Tutino V. Caruso M. G. (2014). Tumor-induced alterations in lipid metabolism. Curr. Med. Chem. 21, 2729–2733. 10.2174/0929867321666140303122426 24606524

[B40] Okubo K. Isono M. Miyai K. Asano T. Sato A. (2020). Fluvastatin potentiates anticancer activity of vorinostat in renal cancer cells. Cancer Sci. 111, 112–126. 10.1111/cas.14225 31675763 PMC6942444

[B41] Oryan A. Kamali A. Moshiri A. (2015). Potential mechanisms and applications of statins on osteogenesis: current modalities, conflicts and future directions. J. Control. Release 215, 12–24. 10.1016/j.jconrel.2015.07.022 26226345

[B42] Otahal A. Aydemir D. Tomasich E. Minichsdorfer C. (2020). Delineation of cell death mechanisms induced by synergistic effects of statins and erlotinib in non-small cell lung cancer cell (NSCLC) lines. Sci. Rep. 10:959. 10.1038/s41598-020-57707-2 31969600 PMC6976657

[B43] Pak E. Segal R. A. (2016). Hedgehog signal transduction: key players, oncogenic drivers, and cancer therapy. Dev. Cell 38, 333–344. 10.1016/j.devcel.2016.07.026 27554855 PMC5017307

[B44] Pisanti S. Picardi P. Ciaglia E. D'Alessandro A. Bifulco M. (2014). Novel prospects of statins as therapeutic agents in cancer. Pharmacol. Res. 88, 84–98. 10.1016/j.phrs.2014.06.013 25009097

[B45] Porter J. A. Young K. E. Beachy P. A. (1996). Cholesterol modification of hedgehog signaling proteins in animal development. Science 274, 255–259. 10.1126/science.274.5285.255 8824192

[B46] Schonewille M. de Boer J. F. Mele L. Wolters H. Bloks V. W. Wolters J. C.. (2016). Statins increase hepatic cholesterol synthesis and stimulate fecal cholesterol elimination in mice. J. Lipid Res. 57, 1455–1464. 10.1194/jlr.M067488 27313057 PMC4959861

[B47] Shiota G. Okubo M. Noumi T. Noguchi N. Oyama K. Takano Y.. (1999). Cyclooxygenase-2 expression in hepatocellular carcinoma. Hepatogastroenterology 46, 407–412. 10228831

[B48] Siegel R. Ma J. Zou Z. Jemal A. (2014). Cancer statistics, 2014. CA Cancer J. Clin. 64, 9–29. 10.3322/caac.21208 24399786

[B49] Siegel R. L. Miller K. D. Jemal A. (2017). Cancer statistic, 2017. CA Cancer J. Clin. 67, 7–30. 10.3322/caac.21387 28055103

[B50] Takai Y. Sasaki T. Matozaki T. (2001). Small GTP-binding proteins. Physiol. Rev. 81, 153–208. 10.1152/physrev.2001.81.1.153 11152757

[B51] Takwi A. A. Li Y. Becker Buscaglia L. E. Zhang J. Choudhury S. Park A. K.. (2012). A statin-regulated microRNA represses human c-Myc expression and function. EMBO Mol. Med. 4, 896–909. 10.1002/emmm.201101045 22887866 PMC3491823

[B52] Vallianou N. G. Kostantinou A. Kougias M. Kazazis C. (2014). Statins and cancer. Anticancer. Agents Med. Chem. 14, 706–712. 10.2174/1871520613666131129105035 24295174

[B53] Wei T. T. Lin Y. T. Chen W. S. Luo P. Lin Y. C. Shun C. T.. (2016a). Dual targeting of 3-hydroxy-3-methylglutaryl coenzyme A reductase and histone deacetylase as a therapy for colorectal cancer. EBioMedicine 10, 124–136. 10.1016/j.ebiom.2016.07.019 27448759 PMC5006731

[B54] Wei T. T. Lin Y. T. Tseng R. Y. Shun C. T. Lin Y. C. Wu M. S.. (2016b). Prevention of colitis and colitis-associated colorectal cancer by a novel polypharmacological histone deacetylase inhibitor. Clin. Cancer Res. 22, 4158–4169. 10.1158/1078-0432.CCR-15-2379 27528734

[B55] Weng C. H. Chen L. Y. Lin Y. C. Shih J. Y. Lin Y. C. Tseng R. Y.. (2019). Epithelial-mesenchymal transition (EMT) beyond EGFR mutations *per se* is a common mechanism for acquired resistance to EGFR TKI. Oncogene 38, 455–468. 10.1038/s41388-018-0454-2 30111817

[B56] Wong W. W. Dimitroulakos J. Minden M. D. Penn L. Z. (2002). HMG-CoA reductase inhibitors and the malignant cell: the statin family of drugs as triggers of tumor-specific apoptosis. Leukemia 16, 508–519. 10.1038/sj.leu.2402476 11960327

[B57] Yang T. Chen M. Sun T. (2013). Simvastatin attenuates TGF-beta1-induced epithelial-mesenchymal transition in human alveolar epithelial cells. Cell. Physiol. Biochem. 31, 863–874. 10.1159/000350104 23817018

[B58] Yin Y. Liu L. Zhao Z. Yin L. Bauer N. Nwaeburu C. C.. (2018). Simvastatin inhibits sonic hedgehog signaling and stemness features of pancreatic cancer. Cancer Lett. 426, 14–24. 10.1016/j.canlet.2018.04.001 29627496

